# Aqueous outflow regulation: Optical coherence tomography implicates pressure-dependent tissue motion

**DOI:** 10.1016/j.exer.2016.06.007

**Published:** 2016-06-11

**Authors:** Chen Xin, Ruikang K. Wang, Shaozhen Song, Tueng Shen, Joanne Wen, Elizabeth Martin, Yi Jiang, Steven Padilla, Murray Johnstone

**Affiliations:** a Department of Bioengineering, University of Washington, USA; b Department of Ophthalmology, University of Washington, USA; c Department of Ophthalmology, Beijing Anzhen Hospital, Capital Medical University, China; d Department of Ophthalmology, Cook County Hospital System, USA

**Keywords:** Glaucoma, Aqueous, Intraocular pressure, Schlemm's canal, Trabecular meshwork, Collector channels, Lymphatics, Pulsatile flow, Optical coherence tomography

## Abstract

Glaucoma is a leading cause of blindness worldwide and results from damage to the optic nerve. Currently, intraocular pressure is the only treatable risk factor. Changes in aqueous outflow regulate pressure; regulation becomes abnormal in glaucoma. From inside the eye aqueous flows out through the trabecular meshwork into a venous sinus called Schlemm's canal, next into collector channels and finally returns to the episcleral vessels of the venous system. The location of aqueous outflow regulation is unknown. *Ex vivo* and *in vivo* studies implicate both pressure-dependent trabecular tissue motion and tissues distal to Schlemm's canal in regulation of aqueous outflow. Technologies have not previously been available to study these issues. New *ex vivo* imaging in human eyes identifies hinged flaps or leaflets at collector channel entrances using a high-resolution spectral domain optical coherence tomography (SD-OCT) platform. The hinged flaps open and close in synchrony with pressure-dependent trabecular meshwork motion. The SD-OCT platform images from the trabecular meshwork surface while experimentally changing transtrabecular pressure gradients. New *in vivo* imaging in human eyes uses a motion sensitive technology, phase-sensitive OCT to quantitate real-time pulse-dependent trabecular tissue motion as well as absence of such motion when aqueous access to the outflow system is blocked. The recent studies suggest that aqueous outflow regulation results from synchronous pressure-dependent motion involving a network of interconnected tissues including those distal to Schlemm's canal. The new imaging technologies may shed light on glaucoma mechanisms and provide guidance in the management of medical, laser and surgical decisions in glaucoma.

## 1. Introduction

### 1.1. Overview

Glaucoma is a leading cause of irreversible blindness in the world and results from damage to the optic nerve (Quigley and Broman, 2006). Intraocular pressure (IOP) is the only treatable risk factor (Heijl et al., 2009). Control of IOP regulation resides within the aqueous outflow system of the eye (Grant, 1958) and IOP regulation becomes abnormal in glaucoma (Grant, 1958; Gabelt & Kaufman, 2011). This article focuses on intrinsic abnormalities of the outflow system in glaucoma in contrast to identifiable extrinsic factors. The intrinsic outflow system abnormality in glaucoma is unknown but is described as primary open angle glaucoma (POAG); the term is reflective of the lack of a clearly identified cause. The term “glaucoma” in this review article will refer only to the enigmatic disease of POAG.

### 1.2. Pathway of aqueous outflow

Aqueous flows from the anterior chamber through the trabecular meshwork (TM) into Schlemm's canal (SC) followed by passage into collector channel entrances (CCE) along SC external wall. From the CCE, aqueous-containing vessels course outward to discharge aqueous to visible episcleral and conjunctival veins on the scleral surface. Because aqueous must flow through these tissues, TM tissue configuration determines aqueous outflow and IOP regulation.

### 1.3. Localized regulation of aqueous outflow

A location between the TM lamellae and the lining of SC inner wall is called the juxtacanalicular space. A prevalent view is that extracellular matrix material (ECM) in the juxtacanalicular space acts like a filter to provide passive resistance to aqueous outflow. Modulation of the properties of the ECM, in conjunction with SC inner wall endothelium interactions, is thought to provide an adjustable resistance. A large body of evidence and carefully reasoned arguments favor the juxtacanalicular space as the primary site of IOP regulation (Gabelt & Kaufman, 2011; Johnstone, 2009). While ECM in the juxtacanalicular space is undoubtedly important and perhaps central to IOP control, imaging evidence suggests additional factors may be involved in controlling aqueous outflow and IOP.

### 1.4. Distributed regulation of aqueous outflow

A passive filter at a single location in the juxtacanalicular space may solely regulate aqueous outflow. If so, there is no need for embarking on the studies of tissue motion described in this review. Section 2 provides a diverse background of evidence pointing to the benefit of exploring features of a more complex regulatory framework. The background evidence points to the benefit of studies of TM and CCE entrance motion. Such studies can help to provide an integrated regulatory model to predict and explain *in vivo* aqueous outflow system behavior in glaucoma.

Evidence from imaging demonstrates pulsatile aqueous outflow and pulse-dependent, pressure-induced physical motion of both the TM and the CCE. *In vivo* imaging studies reveal coordinated, continuously oscillating pressure-dependent pulse waves of aqueous leaving SC (Goldmann, 1946; Ascher, 1942a) and synchronous changes of shape of the pathways through which aqueous flows (Li et al., 2013). Rather than being limited to a single site, aqueous outflow and IOP regulation may result from tensional integration involving subcellular (Johnstone, 2014), cellular (Johnstone, 1979) and tissue-level (Johnstone, 1979, 2004) prestress that permits coordinated synchronous tissue-wide responses.

### 1.5. Article goals

1) To review evidence for the role of distributed tissue-wide pressure-dependent motion in controlling aqueous outflow. 2) To describe a new spectral domain optical coherence (SD-OCT) technology that permits *ex vivo* high resolution imaging of hinged collagen flaps at CCE, a tissue geometry that allows the entrances to open and close. 3) To use the same SD-OCT technology to identify synchronous pressure-dependent TM and CCE motion. 4) To describe a second new imaging technology with high sensitivity to tissue motion, phase-based OCT (PhS-OCT) that permits *in vivo* imaging of pulse-dependent motion of outflow system structures.

## 2. Coordinated proximal and distal tissue motion

### 2.1. Glaucoma surgery points to distal resistance

#### 2.1.1. SC glaucoma surgeries bypass the TM

A series of recently developed minimally invasive glaucoma surgeries (MIGS) bypass the TM providing access to the distal outflow pathways of the eye (Bahler et al., 2012; Johnstone et al., 2014). The procedures are relatively effective, typically lowering IOP to the mid teens (Kaplowitz et al., 2014); risks are modest making the procedures an attractive alternative to glaucoma filtering surgery (Pfeiffer et al., 2015; Grover et al., 2014; Neuhann, 2015). However, it may be argued that the procedures are far from effective in many patients and at times do not lower the pressure more than phacoemulsification alone. Also the procedures do not typically reduce IOP to near episcleral venous pressure (EVP) levels as might be expected if most of the outflow system resistance was in the TM (Richter and Coleman, 2016). Together these findings suggest that the relative ineffectiveness of MIGS needs to be better explained.

#### 2.1.2. IOP levels found after SC surgery suggest distal resistance

The typical post surgery IOP that is achieved (Brandao and Grieshaber, 2013; Saheb and Ahmed, 2012) suggests that resistance distal to SC is important and is probably close to the external wall of SC (Schuman et al., 1999). Perfusion studies also find half or more of the resistance is distal to the TM (Ellingsen and Grant,1972; Rosenquist et al., 1989). In addition, experimental microsurgery (Ellingsen and Grant, 1972; Johnstone and Grant, 1973a; Van Buskirk, 1982) and anatomic studies demonstrate many attachments between the TM (Johnstone, 1974, 2004; Rohen and Rentsch, 1968; Smit and Johnstone, 2002) and the CCE area, suggesting that control of outflow may not be limited to a single site, but rather may be a result of coordinated behavior of connected tissues.

The relative efficacy of the MIGS and yet the inability to explain residual distal outflow resistance point to the need to better understand global tissue mechanics of the outflow system (Loewen and Schuman, 2013), particularly the distal pathways. Rapidly evolving new OCT imaging technology that can image synchronous TM and CCE motion in the laboratory (Li et al., 2013) and trabecular tissue motion in humans in real time (Hariri et al., 2014; Sun et al., 2015) suggest it may be possible to gain a better understanding of intrinsic outflow control mechanisms.

### 2.2. Imaging of pulsatile aqueous outflow abnormalities in glaucoma

#### 2.2.1. Pulsatile aqueous outflow into episcleral veins

**Fig. 1:**
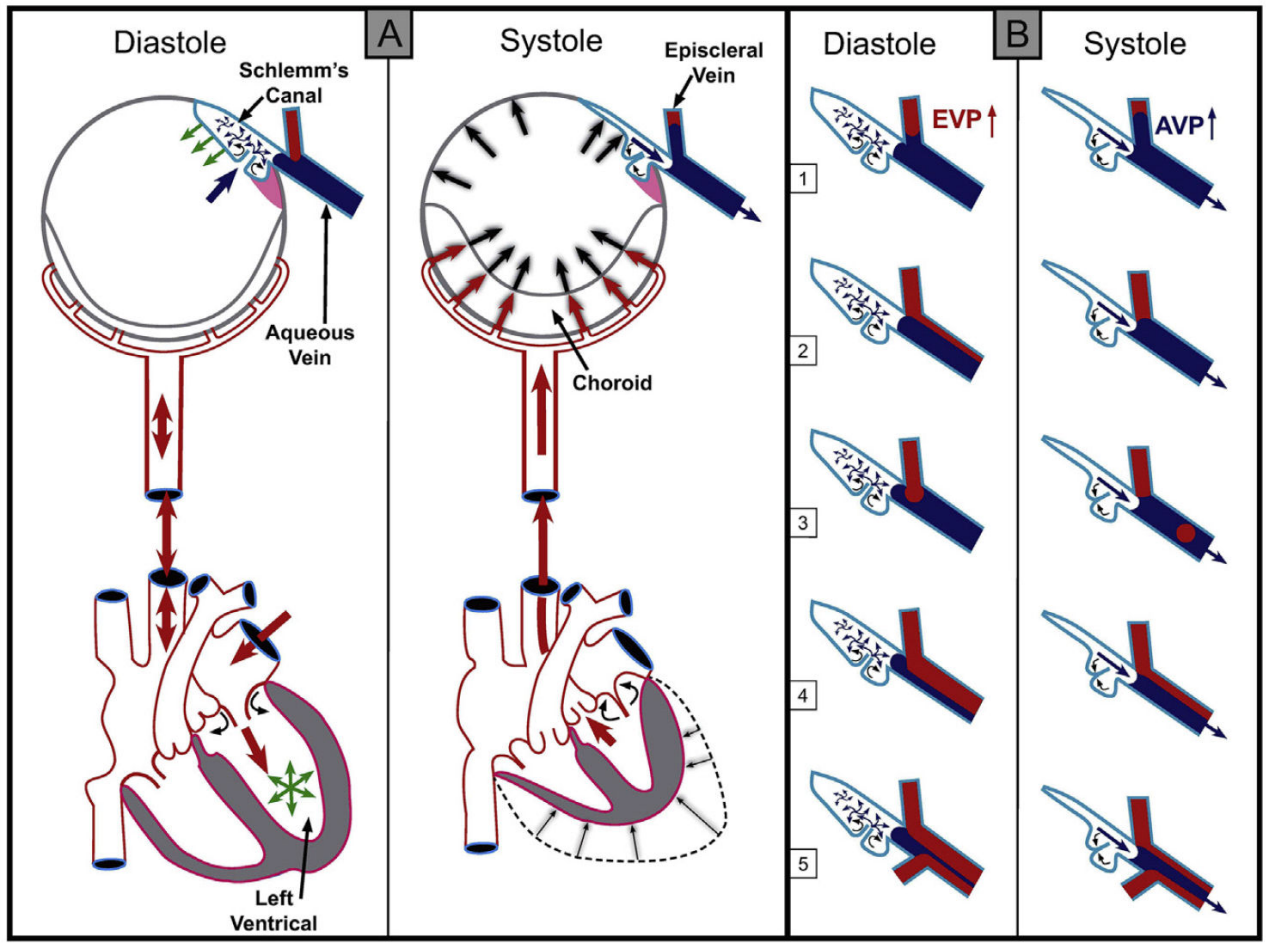
(Panel A-Systole) Cardiac source of pulsatile flow. Systole-induced choroidal vasculature expansion (red arrows). Transient intraocular pressure (IOP) increase (large black arrows). Aqueous pulse wave distends the trabecular meshwork forcing it outward into Schlemm's canal (SC). One-way channels into SC prevent backflow (small curved arrows). Distention of the TM into SC reduces SC volume (black arrows on blue curved surface outlining SC. SC pressure increases. Small black arrow denotes aqueous discharge from SC. Aqueous pulse wave then enters the aqueous vein. (Panel A-Diastole) Blood enters the left ventricle (green circle of arrows). Double red arrows indicate absence of a choroidal pressure wave in diastole. IOP transiently decreases. TM recoils inward during diastole (green arrows). Aqueous enters SC (large blue arrow). (Panel B) During diastole episcleral vein pressure (EVP) is slightly higher than aqueous vein pressure (AVP), resulting in a relative EVP ↑). The EVP ↑ causes episcleral vein blood to move toward or into an aqueous vein. The next systole causes a transient AVP ↑. The oscillations result in pulsatile flow manifestations in the aqueous veins. The AVP ↑ causes transient movement of a standing aqueous wave into a tributary episcleral vein (B1), transient elimination of a lamina of blood (B2), a bolus of blood swept into the increased aqueous stream (B3), an oscillating increase in diameter of the aqueous component of a persistent laminar (B4) or a trilaminar (B5) aqueous flow wave. From: Johnstone M, Jamil A, Martin E. Aqueous Veins and Open Angle Glaucoma. In: Schacknow P, Samples J, editors. The Glaucoma Book. New York: Springer, 2010. p. 65–78.

**Fig. 2:**
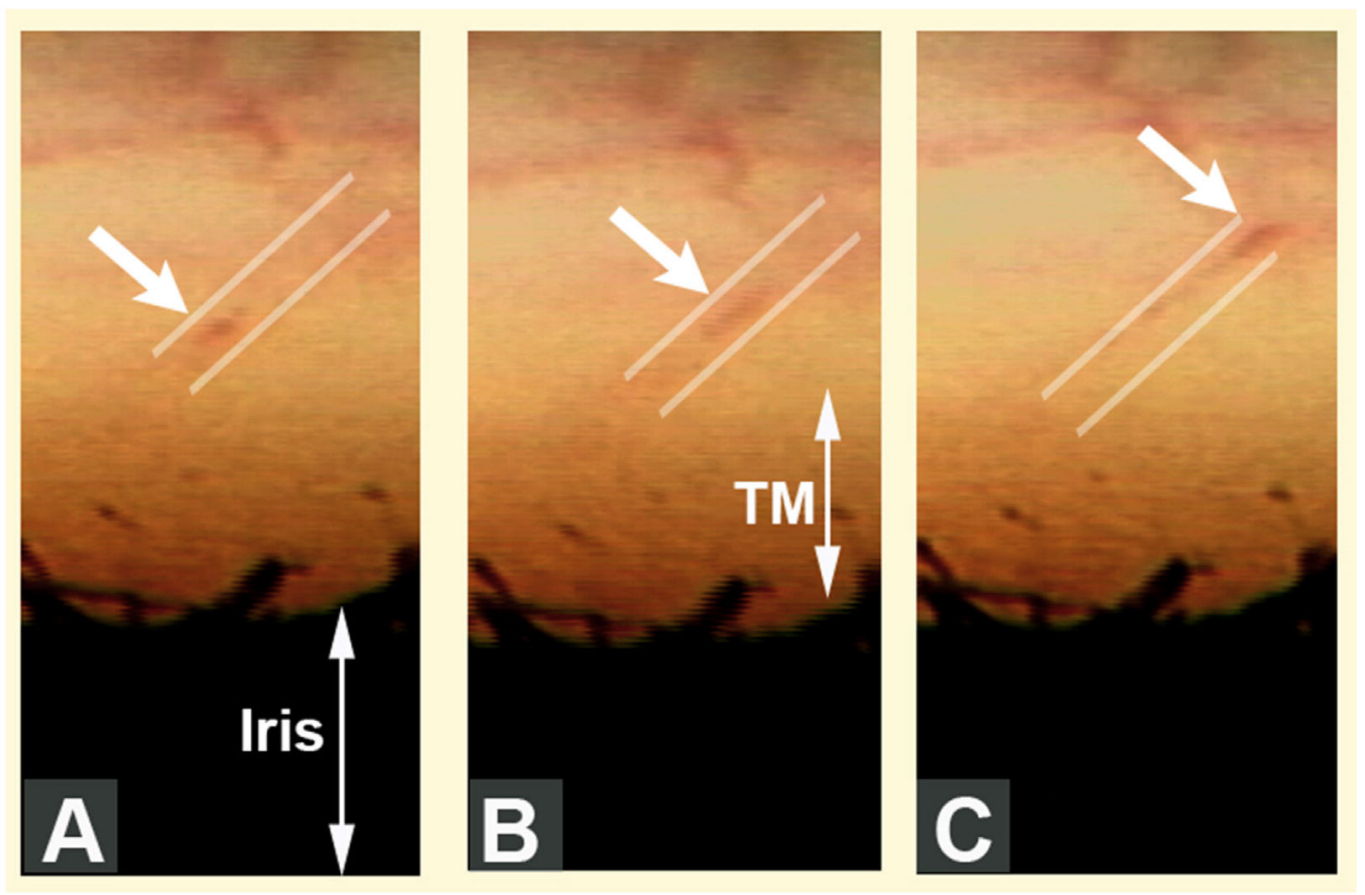
Pulsatile aqueous movement along a collector channel viewed *in vivo* with a gonioscopy lens. Parallel white lines above the area of the trabecular meshwork (TM) depict the course of movement of blood-tinged aqueous (*white arrows*) along an aqueous vein in sequential video frames encompassing one systolic pulse wave. **A**–**C** (Gonioscopic video courtesy of R. Stegmann). From: Johnstone MA. A New Model Describes an Aqueous Outflow Pump and Explores Causes of Pump Failure in Glaucoma. In: Grehn H, Stamper R, editors. Essentials in Ophthalmology: Glaucoma II. Heidelberg: Springer, 2006.

Reports of the discovery of pulsatile aqueous outflow and aqueous vein identification occurred simultaneously; the pulsatile nature of aqueous outflow was a salient feature discussed in the original papers as a means of aqueous vein recognition (Goldmann, 1946; Ascher, 1942a). Pulsatile outflow originates from SC and is synchronous with the ocular pulse (Fig. 1) (Ascher, 1961; Johnstone et al., 2010). The ocular pulse in turn results from changes in choroidal volume that occur with the cardiac cycle (Phillips et al., 1992). Video imaging demonstrates pulse-dependent patterns of aqueous outflow from SC into CCE (Johnstone, 2006) (Fig. 2). Directly verifiable video imaging also provides quantitative measurements of the volume of the pulse waves of aqueous entering the aqueous veins (Stepanik, 1954).

#### 2.2.2. Pulsatile aqueous outflow abnormalities identify glaucoma and its severity

Pulsatile outflow from SC into the aqueous veins is altered in glaucoma patients (Ascher, 1961, 1949, 1953). For example, one study noted pulsatile flow in 196 aqueous veins of 111 normal subjects but in only 6% of glaucoma patients. No pulsatile flow was noted in patients with an IOP higher than 28 mm Hg. Pulsatile aqueous outflow issues in the subset of patients with normal pressure glaucoma is yet to be systematically studied.

The compensation maximum test uses ophthalmodynamometry to increase IOP while imaging aqueous veins (Kleinert, 1951; Stambaugh et al., 1954). In normal subjects pulsatile outflow increases as IOP increases while in glaucoma patients, pulsatile outflow slows or stops (Ascher, 1961; Johnstone, 2006). In the aqueous influx test, distal compression of an aqueous vein results in an increase in pulsatile outflow in the proximal aqueous veins in normal subjects (Ascher, 1942b, 1944; De Vries, 1947). In glaucoma, pressure on distal episcleral veins causes proximal veins to fill with blood (Ascher, 1961; Goldmann, 1948; Thomassen, 1949).

In glaucoma patients with reduced or absent pulsatile outflow, outflow medications that reduce IOP increase pulsatile aqueous outflow; medications include miotics (Ascher, 1942a, 1942c; De Vries, 1947; Thomassen, 1947; Cambiaggi, 1958), adrenergics (Ascher, 1942a, 1942c; De Vries, 1947; Thomassen, 1947; Cambiaggi, 1958) and prostaglandins (Johnstone et al., 2011). Pulsatile aqueous outflow improvement is made manifest by an increase in amplitude, speed and length of distal aqueous pulse wave progression along the vein. Beyond the duration of the effect of the medications, pulsatile outflow again returns to its diminished or absent status. Aqueous pulse waves induced at surgery have been found to have potential use in predicting the success of SC microsurgery (Fellman and Grover, 2014).

### 2.3. SC blood reflux reveals TM motion abnormalities in glaucoma

#### 2.3.1. Normal physical activity results in SC pressure gradient reversal

Physiologic activities such as gymnastics and yoga commonly involve body inversion. The pressure reversal in the systemic vasculature causes a rise in episcleral venous pressure (EVP). The rise in EVP causes the pressure in SC to also become higher than IOP causing a reversal of pressure gradients across the TM (Weinreb et al., 1984). The pressure reversal in SC causes blood to enter the canal (Friberg et al., 1987). Aqueous in the anterior chamber cannot flow against the higher pressure in SC; a highly significant correlation has been found between EVP and the IOP rise associated with increased SC pressure (Friberg et al., 1987). For every mm Hg rise in EVP, there is thought to be an almost equal rise in IOP.

Two studies reported an IOP increase within seconds from the mid-teens to mid-30s following inversion (Weinreb et al., 1984; Friberg and Weinreb, 1985). Following inversion, IOP and SC pressures rise to as high as 43 mm Hg (Friberg et al., 1987). Syndromes involving venous obstruction and arteriovenous anomalies can cause marked EVP and SC pressure elevation that prevents aqueous from leaving the anterior chamber. The resulting EVP-dependent IOP elevation can lead to intractable glaucoma (Phelps, 1978; Phelps et al., 1982).

#### 2.3.2. SC pressure reversals moves the TM far away from SC external wall

The canal is little more than a potential space at physiologic pressures (Johnstone and Grant, 1973b; Grierson and Lee, 1975a). Intentional SC pressure gradient reversal used clinically causes blood to reflux into and dilate SC (Kronfeld et al., 1942). *In vivo* studies in primates demonstrate that with as little as a 4 mm reversal of pressure gradients the TM moves far from SC external wall and the TM collapses resulting in a widely dilated canal lumen (Johnstone et al., 1980). Gonioscopy is a clinical technique that places a special lens on the surface of the eye. The lens permits direct imaging of the TM under high power magnification of a slitlamp. Use of a suction or flanged goniolens as well as aqueous aspiration intentionally causes pressure to be higher in SC than in the anterior chamber.

#### 2.3.3. SC pressure reversal identifies glaucoma and its severity

The TM is transparent, permitting direct observation and imaging of blood entering SC. In normal eyes direct imaging reveals that SC pressure reversal causes blood to rapidly fill the lumen of SC; with cessation of pressure reversal SC rapidly empties. In mild glaucoma, SC fills and empties slowly, in more advanced glaucoma there is very slow patchy SC filling. In far advanced glaucoma, there is a complete absence of entry of blood into SC (Kronfeld, 1949; Suson and Schultz, 1969; Schirmer, 1969, 1971; Phelps et al., 1972). Poor filling of SC following SC pressure reversal in the operating room is predictive of failure of canaloplasty, which is a form of SC glaucoma microsurgery (Grieshaber et al., 2010).

### 2.4. Laboratory studies characterize loss of TM motion in glaucoma

#### 2.4.1. Perfusion studies characterize tissue level TM motion

**Fig. 3:**
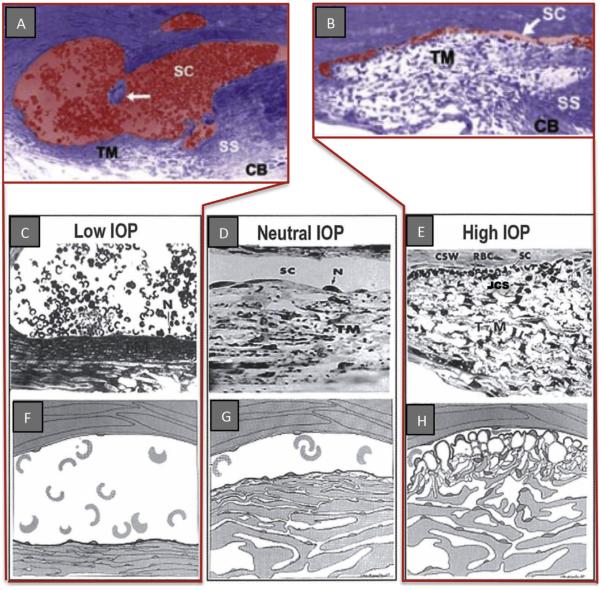
Pressure-dependent trabecular meshwork (TM) configuration. A and B are micrographs of two eyes of the same primate with eyes fixed simultaneously *in vivo*. (A, C, & F); Low IOP; IOP = 0 mm Hg, episcleral venous pressure ~ 8 mm Hg. The scleral spur (SS) is rotated inward toward the anterior chamber. The lumen of Schlemm's canal (SC) is large; the TM tissues are collapsed with obliteration of the juxtacanalicular space (JCS). (**D, G**) (Neutral lOP) IOP = 0, EVP = 0 the trabecular tissues are in a neutral position, SC lumen size is modest. (**B, E, H**) (High lOP) lOP = 25 mm Hg, EVP ~ 8 mm Hg during fixation; IOP reduced to 0 mm Hg after fixation with animal still alive. Blood refluxes into SC. The SS is rotated toward SC. The lumen of SC is reduced to a potential space. SC inner wall endothelium distends to reach Schlemm's canal external or corneoscleral wall (CSW). The JCS is large. Large spaces are present between the trabecular lamellae. Red blood cells (RBC) are present in SC but not the JCS. (N, nucleus of Schlemm's canal endothelial cell.) (RBC dimensions ~ 8 μm) From: Johnstone MA. Grant WM: Pressure-dependent changes in structure of the aqueous outflow system in human and monkey eyes, Am J Ophthalmol 75:380, 1973.

By fixing eyes *in vivo* at a series of systematically controlled perfusion pressures, steady state pressure-dependent TM responses have been characterized (Johnstone, 1974; Johnstone and Grant, 1973b; Grierson and Lee, 1975a). In living eyes with normal EVP but IOP below EVP, the TM collapses into a solid sheet of tissue and the SC lumen is widely dilated (Johnstone, 1974; Johnstone and Grant, 1973b; Johnstone et al., 1980; Grierson and Lee, 1975b). At increasing pressures, the TM distends progressively into SC and at physiologic pressures begins to develop progressive apposition to SC external wall (Fig. 3) (Grierson and Lee, 1975b). The tissue-level configuration changes resulting in SC closure are consistent with conclusions from earlier studies of Grant and colleagues doing perfusion studies (Ellingsen and Grant, 1971, 1972) as well as later lens depression studies of Van Buskirk (Van Buskirk, 1982; Van Buskirk, 1976).

#### 2.4.2. Perfusion studies characterize cellular level TM motion

At the cellular level, the cells of the entire SC inner wall move outward. Individual SC endothelial cells, where untethered by cell processes to underlying trabecular lamellae, move outward into SC, forming large pseudovacuoles (Johnstone, 1979; Johnstone and Grant, 1973b; Grierson and Lee, 1975a). Juxtacanalicular cells and processes deform at the process origins consistent with the presence of cellular stress (Johnstone, 1979; Johnstone and Grant, 1973b; Grierson and Lee, 1974a; Grierson et al., 1978). As pressures increase further, the TM becomes appositional to SC external wall preventing aqueous access to CCE (Johnstone and Grant, 1973b; Grierson and Lee, 1975b). Endothelial-lined cylindrical regions arising from SC inner wall also move into CCE (Grierson and Lee, 1974b); a similar finding occurs in the aqueous plexus of bovine eyes with elevated IOP (Battista et al., 2008).

#### 2.4.3. Perfusions demonstrate TM motion causes resistance in glaucoma

Ellingsen and Grant compared two anterior chamber perfusion conditions; first with iridectomy so that the lens remained in a neutral position; second without iridectomy where anterior chamber perfusion caused reverse pupillary block. The reverse pupillary block caused the lens-iris diaphragm to move backward. Backward lens motion exerted tension on the zonules and ciliary body. The increasing tension on the attached trabecular tendons and on the scleral spur caused the TM to move away from SC external wall. Chamber deepening caused a profound reduction in resistance, completely eliminating the increasing resistance previously found with increasing IOP (Ellingsen and Grant, 1971). More importantly, the effect of tension causing SC wall separation was far more pronounced in glaucoma eyes. The effect of chamber deepening that separates SC wall and eliminates increasing resistance with increasing IOP points to a physical relationship between TM movement, SC lumen enlargement and resistance to aqueous outflow.

The finding that increasing resistance with increasing IOP is far more pronounced in glaucomatous eyes is consistent with the concept that SC wall apposition is a factor in the resistance issue in glaucoma (Ellingsen and Grant, 1971). More importantly, the findings suggest that if a therapeutic approach could be found that places tension of the scleral spur and opens SC, the resistance issue in glaucoma might be reversible. Of interest, pilocarpine does achieve the above anatomic aim (Johnstone, 2009). For the duration of the pilocarpine action, the glaucoma abnormality also appears to be functionally reversible. Pulsatile aqueous outflow improves (Ascher, 1942c, 1961; Thomassen, 1947; Cambiaggi, 1958) indicative of an improved ability of the outflow tissues to move. The increased pulsatile flow is followed by a reduction in IOP that persists until the pharmacologic effect of pilocarpine disappears (Ascher,1942c,^1961^).

#### 2.4.4. Perfusion studies demonstrate locations of motion-dependent resistance

Experimental perfusion in *ex vivo* eyes by Grant and colleagues suggest that perhaps 50% of resistance or less can be attributed to the TM (Ellingsen and Grant, 1972; Rosenquist et al., 1989; Johnstone and Grant, 1973a; Ellingsen and Grant, 1971). Studies by Van Buskirk used lens depression to place tension on the zonules (Van Buskirk, 1976; Van Buskirk and Grant, 1973). The tension transmitted through the ciliary body places tension on the TM rotating it away from SC external wall. Van Buskirk histologically demonstrated progressive movement of trabecular lamellae away from each other, movement of SC inner wall endothelium away from the lamellae to enlarge the juxtacanalicular space, and movement of SC endothelium away from SC external wall to markedly enlarge SC lumen (Van Buskirk, 1982).

There was a corresponding highly linear reduction in outflow resistance associated with lens depression that rotates the scleral spur inward with a resulting separation of the walls of SC (Van Buskirk, 1982). The group of studies points to TM motion as being pressure sensitive and an important factor in outflow resistance. It also suggests the need to maintain tightly controlled elasticity and compliance of the TM tissues to optimize spacing between the TM lamellae, between the TM lamellae and SC inner wall endothelium (the juxtacanalicular space) and particularly the relationships between SC walls.

### 2.5. Laboratory evidence of tissue and cell stiffening in glaucoma

#### 2.5.1. Pulsatile aqueous outflow, cell deformation and shear stress relationships

Regulation of TM motion is thought to become impaired in glaucoma. Evidence includes a reduced ability of SC lumen to change dimensions and diminished pulsatile aqueous outflow in aqueous veins; both phenomena are thought to have tissue stiffening as an underlying cause. The cellular components of the TM, particularly the individual SC endothelium and juxtacanalicular cells, move and deform in response to IOP changes within the physiologic range (Johnstone, 1979; Johnstone and Grant, 1973a; Grierson and Lee, 1975a). The ability to sense environmental stimuli such as IOP fluctuations and cyclic IOP oscillations results when pressure-dependent, tensionally integrated cells undergo deformation and experience shear stress (Ingber, 2008).

Cellular deformation responses associated with pulse-induced motion are dependent on the properties of intracellular contractile machinery. Alterations of the TM contractile state have demonstrated that a reduction of actomysin contractility is associated with an expansion of spaces of the TM in human tissue (Gonzalez et al., 2016). Recently these mechanisms have been probed *in vivo* in a living mouse model where actomysin inhibition also has been shown to modulate outflow resistance (Ko et al., 2016). Cochlin has been shown to be capable of regulating IOP (Goel et al., 2012), to modulate trabecular cell elongation (Goel et al., 2011), to be associated with glaucomatous TM (Bhattacharya et al., 2005), and to also have a role in mechanosensing of shear stress (Goel et al., 2012).

#### 2.5.2. Cyclic stresses, gene expression, cytoskeletal networks, signal transduction

In an *ex vivo* model, cyclic changes in IOP are found to alter conventional aqueous outflow (Ramos and Stamer, 2008). Because a synchronous increase in cellularity associated with cytoskeletal contractile machinery occurs, it is proposed that cyclic changes act to alter cellular contractile mechanisms (Ramos et al., 2009). Such findings have led to the proposal that a physiologic benefit important to IOP homeostasis is conferred by the cyclic mechanical stresses. A need to improve our understanding of the linkage between cyclic stresses, TM motion, and IOP regulatory homeostasis is suggested by these studies.

Mechanical stresses lead to alterations in gene expression (Luna et al., 2009a; Liton et al., 2005; Tumminia et al., 1998; Tamm et al., 1999), changes in cytoskeletal networks (Junglas et al., 2012; Mitton et al., 1997) and modulation of signal transduction (Luna et al., 2009b). Cells of the outflow system determine extracellular matrix (ECM) composition. Mechanical stretching modulates the composition of the ECM in the trabecular beams and juxtacanalicular space (Bradley et al., 2003; Vittal et al., 2005). Elasticity and compliance of the TM is in turn controlled by ECM composition. Modulation of ECM composition becomes abnormal in glaucoma (Keller et al., 2009; Stamer and Acott, 2012).

#### 2.5.3. Tissue stiffening

Studies implicate changes in cell and ECM stiffness as probable factors in the glaucoma process (Filla et al., 2011; Clark et al., 2013). A relationship between increased TM tissue stiffness and open angle glaucoma is found in recent elastic modulus determination studies (Last et al., 2011; Raghunathan et al., 2013; Thomasy et al., 2013); the change in tissue stiffness properties is also identified in clinical research studies (Johnstone, 2006; Suson and Schultz, 1969; Schirmer, 1969). Studies involving steroids (Raghunathan et al., 2015) and the Wnt pathway (Morgan et al., 2015) further implicate increased stiffness of cells and ECM of the TM tissues as factors involved in the outflow regulation failure in glaucoma.

### 2.6. IOP and CCE motion mediated by TM attachments

#### 2.6.1. Connections between the TM and SC external wall may hold CCE open

**Fig. 4:**
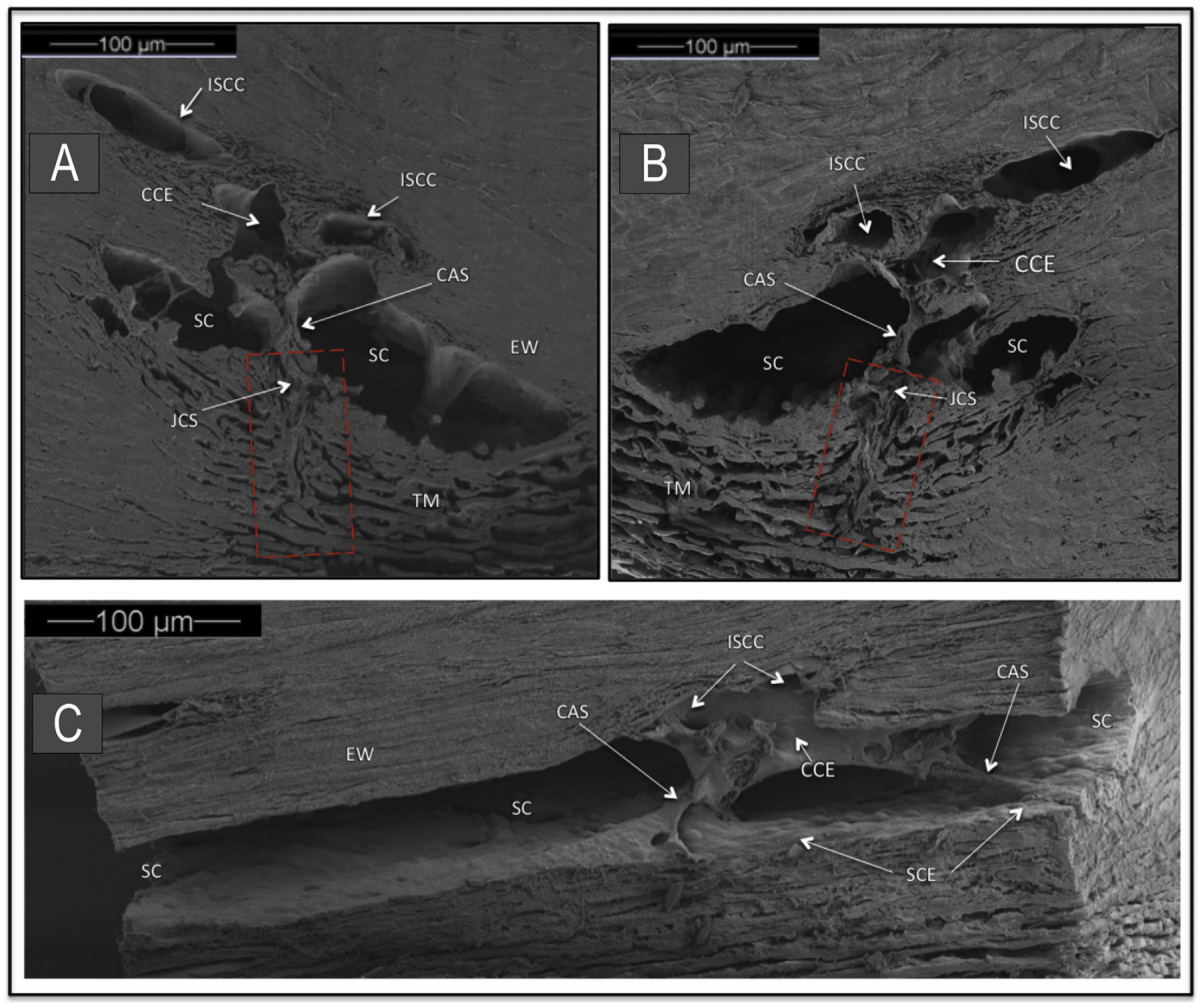
Organization of aqueous outflow pathways. Scanning electron microscopy (SEM) of a macaca nemestrina monkey eye. (**A, B**) are paired mirror image radial sections of trabecular meshwork (TM), Schlemm's canal (SC) and collector channel ostia (CCO). A cylindrical attachment structure (CAS) has a lumen that communicates with the juxtacanalicular space (JCS) and the collector channel entrance (CCE). Red rectangle indicates TM region organized parallel to probable path of preferential aqueous flow into large open funnel at origin of a CAS. Intrascleral collector channel (ISCC). SC external wall (EW). (**C**) SEM section parallel to the circumference of SC. CAS span across SC to hinged flaps at CCE. A direct path for aqueous flow from SC into the CCE and ISCC is visible.

Studies by Rohen and Rentsch (Rohen and Rentsch, 1968) were the first to point out that the organization of tissues surrounding the CCE permits them to move and that the motion may be capable of altering CCE lumen dimensions. Their studies further observe that structures arising from the TM span across SC and connect to the regions of CCE. They conclude that the configuration of the structures connecting the TM and SC may help to hold the CCE open as pressure increases. Fig. 4 demonstrates attachments between the TM and CCE as originally described by Rohen and Rentsch (Rohen and Rentsch, 1968).

In support of the findings of a correlation between pressure-dependent TM motion and CCE relationships, a micro computerized tomography (CT) study (Hann et al., 2011) reports that the CCE size is markedly different in an immersion fixed eye (27.5 μm) compared with a perfused eye fixed at 10 mm Hg (40.5 μm). In another perfusion study (Hann et al., 2014) a 3.7-fold increase in CCE occlusion was found in glaucoma compared with normal eyes. In glaucoma eyes, SC volume, CCE area and CCE diameter were decreased compared to normal eyes at like pressures.

#### 2.6.2. The lymphatic connection

Schlemm's canal has recently been shown to be a lymphatic-like vessel (Aspelund et al., 2014). The aqueous outflow system acquires lymphatic identity through upregulation of the master lymphatic regulator PROX1. As a result, SC tissues express the lymphatic valve marker FOXC2 but also integrin alpha9, continuous vascular endothelial cadherin junctions and basement membrane features like the collecting lymphatics (Park et al., 2014). Recently, disruption of genetic mechanisms associated with lymphatic development has been shown to be present and to cause defective development of outflow pathways leading to glaucoma in a laboratory model (Thomson et al., 2014). These linkages between abnormal lymphatic developmental signals and glaucoma suggest that like the lymphatics, pulsatile behavior may be an important factor in movement of aqueous. In addition, imaging of abnormalities of pulsatile behavior may yield information important to understanding the glaucoma process.

Extracellular fluid homeostasis is tightly controlled by regulation of return of lymphatic fluid to the vascular. Although once viewed as passive conduits like arteries, lymphatic vessel segments between valves exhibit structural behavior in common with ventricles (Quick et al., 2007, 2009). It is well established that they can actively pump lymph against an axial pressure gradient from low-pressure tissue to the great veins of the neck (Quick et al., 2009). When outlet pressure falls below inlet pressure they can transition from pump to conduit behavior (Quick et al., 2007, 2009).

Lymph flow is regulated by pulsatile unidirectional flow requiring tissue motion and valves. Aqueous and lymph both return to the vascular system by such unidirectional pulsatile mechanisms. A lymphatic-like model has been proposed to explain pulsatile aqueous outflow (Johnstone, 1974, 2004) and failure of pulsatile outflow in glaucoma (Johnstone, 2006). A chokepoint distal to SC that minimizes aqueous backflow is useful to provide a more satisfactory model. The findings of CCE connections to the TM as noted in Section 2.6.1 above and recent evidence that the CCE undergo synchronous changes in shape (Hariri et al., 2014) as discussed in Section 4.5 below suggest the presence of such functional distal chokepoints.

## 3. TM motion identification with commercial OCT

### 3.1. Motion identified with commercial OCT

SC lumen dimensions are dependent on and are reflective of TM motion. For example, *in vivo* experimental studies demonstrate that when IOP increases, SC lumen becomes smaller resulting from TM distention into SC (Johnstone and Grant, 1973b; Grierson and Lee, 1975a). OCT imaging permits dynamic noninvasive real-time assessments of living tissue (Huang et al., 1991; Fercher et al., 2003; Tomlin and Wang, 2005). The technology provides high-resolution (<10 μm) and high-speed imaging of three-dimensional (3- D) tissue structures making the OCT a potentially valuable tool for studying the outflow system in normal and glaucoma eyes.

**Fig. 5:**
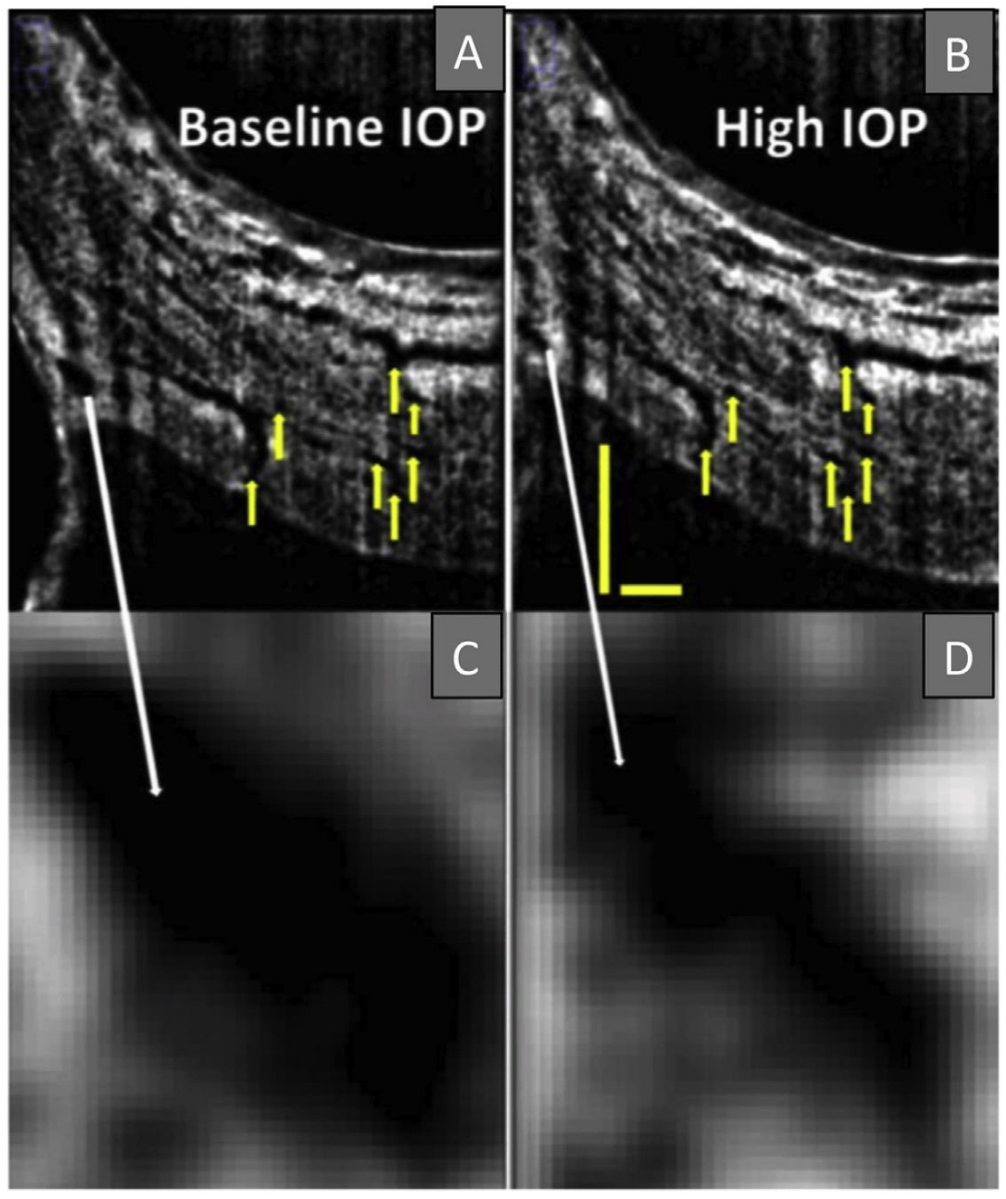
B-scans of Schlemm's canal at baseline (A) and during acute IOP elevation (B) matched based upon vascular landmarks (yellow arrows). Magnification reveals a decrease in Schlemm's canal cross-sectional area from baseline (C) to high IOP (D). Scale bars: 500 μm (scans have an anisotropic aspect ratio, and for that reason the horizontal and vertical scale bars are of different lengths). (For interpretation of the references to colour in this figure legend, the reader is referred to the web version of this article.)

Commercially available OCT instruments have made strides in assessing TM motion associated with changes in SC dimensions. A correlation has been shown between cross-sectional SC area and IOP in several studies; the results are consistent with findings from a morphometric analysis system showing pressure-dependent movement in *ex vivo* human and nonhuman primate (NHP) eyes (Johnstone and Grant, 1973b). The landmark study of Kagemann and colleagues demonstrates that SC area decreases in response to an acute change in IOP in healthy human subjects (Kagemann et al., 2014) (Fig. 5). Furthermore, SC area is significantly smaller in glaucomatous compared to normal eyes (Kagemann et al., 2010; Wang et al., 2012).

### 3.2. Limitations in assessing TM motion with commercial OCT systems

Challenges remain in using OCT imaging technology in the aqueous outflow system. It remains difficult to quantify SC dimensions or characterize structures spanning across SC. In addition, image resolution has been insufficient to delineate details of CCE and their relationships to intrascleral collector channels in the deep scleral plexus. Of greater concern has been the inability to characterize the time scale of TM motion in these tissues, particularly tracking of pulse-dependent motion.

Scattering from the limbal tissue and shadowing from the vasculature above SC reduces the effective power of the imaging beam reaching the canal. These factors diminish image contrast, preventing accurate delineation of the borders of the canal while also precluding characterization of structures within the canal, structures surrounding CCE and relationships in the deep scleral plexus (Kagemann et al., 2010). When longer wavelengths are used to enhance light penetration into the tissue, image contrast improves but axial resolution diminishes (Usui et al., 2011).

An endoscopic OCT probe to image the outflow system from the TM surface circumvents the scattering and shadowing problems, permitting identification of CCE (Ren et al., 2011). However, the imaging rate of the probe of 0.5 frames per second prevents it from being useful in assessing TM or CCE motion in response to cyclic pulse-induced variations of IOP.

*In vivo* studies have inevitable motion artifact. Comparative studies are problematic because it is difficult to repeatedly image the same location. The lumen of SC is also normally little more than a potential space with the TM in close approximation to SC external wall creating a particularly difficult additional challenge for assessing TM-CCE relationships (Johnstone and Grant, 1973b; Grierson and Lee, 1975a). An additional problem results from the inability to study relationships of structures or characterize the motion of structures connecting the TM and the scleral flaps surrounding CCE.

## 4. *Ex vivo* SD-OCT high-resolution platform for structure and motion detection

### 4.1. The technology

SD-OCT systems are pixel based and capable of an optimized image resolution in the range of <4 μm if designed properly. Images are of structure only. Motion can be characterized with SD-OCT by analyzing serial time-series structural frames but only if resolution is high and intervals between successive frames is short. Patient motion, scleral scattering and vessel shadowing prevent SD-OCT systems from obtaining a high resolution of the SC and collector channel region *in vivo*. In addition, the poor signal to noise ratio prevents studies of rapid pulse-dependent motion. We recently reported studies that circumvented these issues by using a stable *ex vivo* preparation that images the outflow system from the trabecular meshwork rather than the scleral surface (Hariri et al., 2014).

The absence of motion or vessel shadowing along with greatly reduced scattering provided a much-improved signal to noise ratio and sufficiently high resolution to image details of SC and CCE structural relationships. SD-OCT imaging while simultaneously changing transtrabecular pressures permitted identifying time-dependent changes in structural images of SC and the CCE. Such time-dependent measurements permit determining pulse amplitude-dependent tissue velocities and displacement.

### 4.2. Crucial guidance for ex vivo experimental design provided by in vivo studies

Because of a normal TM configuration that places the meshwork close to SC external wall and because of the scleral thickness, it has not been possible to provide high resolution imaging of real time SC and CCE entrance motion; yet many studies point to the importance of gaining an understanding of the motion of this region.

Knowledge gained from eyes of normal and glaucoma patients provides a clear rationale for the use of the laboratory-based studies to be described below. Section 2.3.1 discusses the fact that EVP varies markedly under physiologic conditions. A physiologic pressure reversal and blood reflux into SC occurs when EVP pressure is higher than IOP. Various degrees of body inversion result from many physiologic activities that then lead to reversal of pressure gradients with attendant blood reflux into the canal. In association with blood reflux EVP, SC pressure and IOP can rise into the 40s.

Clinical use of reversal of SC pressure gradients with blood reflux into SC is reported in the classic outflow system studies that stratify normal, glaucoma suspects and glaucoma patients discussed in detail in Section 2.3.3; along with pulsatile outflow changes these are the only type of studies that provide evidence of directly verifiable outflow system abnormalities in glaucoma. The technique of SC pressure reversal is also used in clinical research studies to predict who will have success following a SC glaucoma procedure as noted in Section 2.3.3. Knowledge of the effects of *in vivo* SC pressure reversal developed through systematic physiology studies provides a rational and intuitive foundation for use of the technique to be described in the following section.

### 4.3. Ex vivo SD-OCT platform components

*A* recently reported experimental OCT system (Hariri et al., 2014) circumvents the imaging limitations and challenges outlined in Section 3.2. Segments of the outflow system of *ex vivo* non-human (NH) primate eyes were imaged from the TM surface using a high-resolution OCT system with a spatial resolution approaching 4 μm (Hariri et al., 2014). The setup avoids scleral light scattering and vessel shadowing as well as eliminating motion artifact. In addition, the *ex vivo* environment eliminates the imposing limitations presented by *in vivo* tissue motion artifacts.

A cannula attached to a perfusion reservoir is inserted into SC. Reservoir height adjustments permit maintaining steady state pressures in SC. Switching between perfusion reservoirs at different heights provides a means to track instantaneous pressure-dependent motion. The system permits quantitative assessment of TM, SC and CCE structural relationships under steady state conditions using both 2D and 3D modalities. Continuous 2D OCT imaging while switching between reservoirs permits real time tracking of pressure-dependent tissue motion.

Because SC is often little more than a potential space preventing satisfactory delineation of SC and CCE walls with OCT imaging, the current SC perfusion technique makes use of SC dilation to permit far more clear delineation of relationships. The experimental platform mirrors the clinical studies that have been used so successfully in identifying outflow system abnormalities in glaucoma as outlined in Section 2.3. With this *ex vivo* system, steady-state pressure conditions permit measurement of TM tissue responses under controlled conditions as well as reconstruction of SC volumes with a much higher resolution than that obtained with transcleral measurements. The lumen of SC, CCE and structures surrounding CCE can be easily identified. Both structural element motion and resultant lumen dimension changes can be quantified.

## 5. *Ex vivo* SD-OCT images pressure-dependent position of hinged flaps at CCE

The study by Rohen and Rentsch (Rohen and Rentsch, 1968) is the first to describe hinged collagen flaps at CCE entrances. The study notes the hinged flaps are attached at only one end and thus are free to move. Their study also describes connections between the TM and the CCE hinged flaps. The study furthermore points out that the connections are necessary to hold CCE open sufficiently to allow aqueous flow.

### 5.1. Ex vivo steady-state measurements of hinged flap pressure-dependent position

**Fig. 6:**
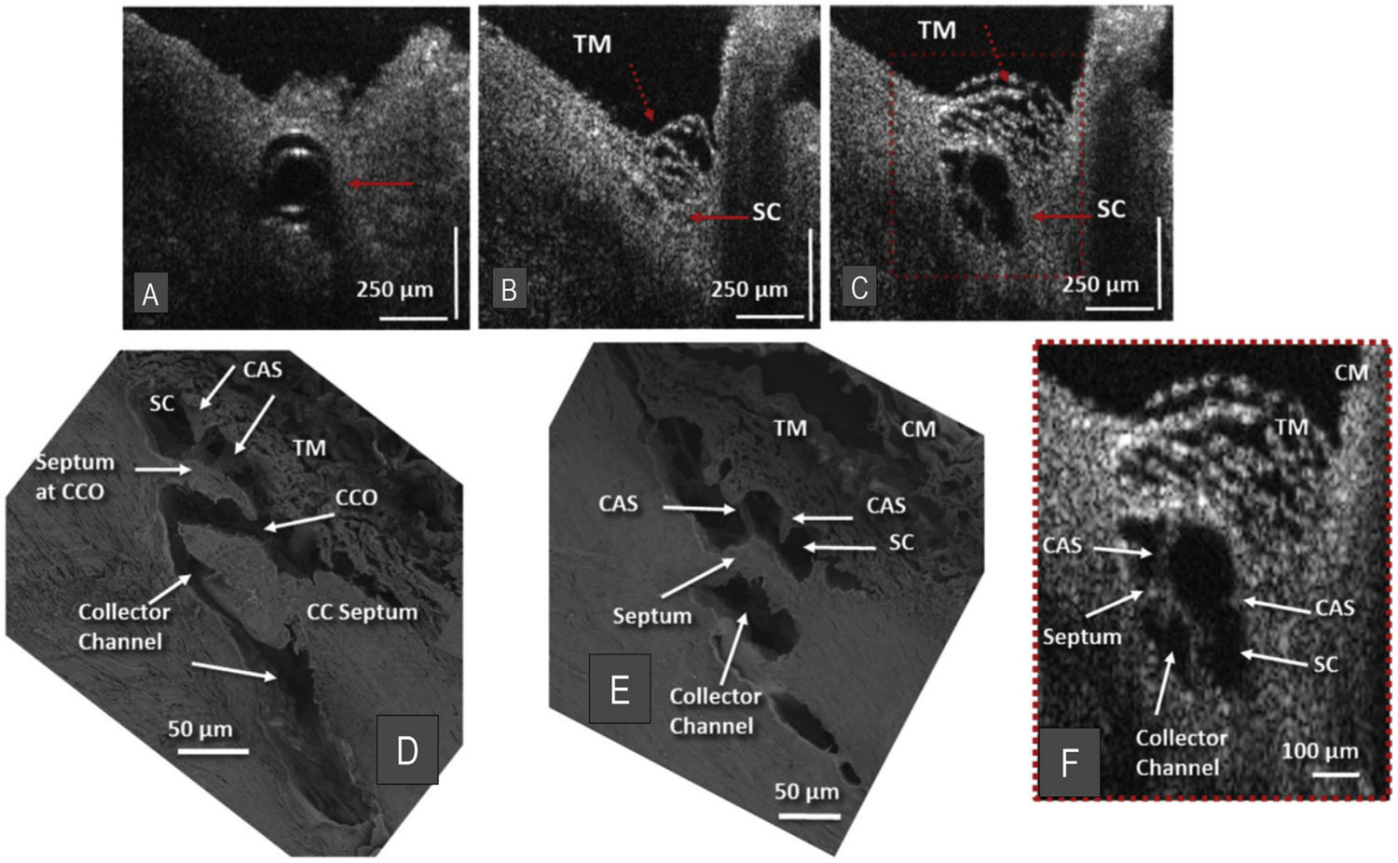
Representative two-dimensional (2-D) structural OCT images from the limbal region of a nemestrina monkey eye. (A) shows a perfusion cannula inside Schlemm's canal (SC) (red arrow). (B) Red arrows identify trabecular meshwork (TM) and SC location ~150 μm beyond the cannula tip before introduction of perfusate. (C) shows the maximally dilated appearance of SC at the same location as in (B) following introduction of perfusate. Video available at (www.youtube.com/watch?v=QhN4yJAzYeY) showing transition from (B) to (C). (D) is a scanning electron microscopy (SEM) image of the outflow system of a nemestrina primate eye showing a collector channel entrance or ostia (CCO) that communicates with an intrascleral collector channel that turns circumferentially in SC. Cylindrical attachment structures (CAS) provide connections between the TM and a septum that creates a hinged flap at the CCO. (E) is the SEM section adjacent to the image in D showing how the CCO region transitions from a channel communicating with SC to a circumferentially oriented intrascleral collector channel. (F) is the 2× enlargement the OCT image cropped from (C) (red dashed box) and permits a more detailed comparison of relationships. Structural features of the outflow system are mirrored in both the SEM images of the eye (D & E) and the enlarged OCT image (F) that illustrate SD-OCT resolution approaching that of SEM. Original SEM images: 337× magnification. CM, ciliary muscle. From: Hariri S, Johnstone M, Jiang Y et al. Platform to investigate aqueous outflow system structure and pressure-dependent motion using high-resolution spectral domain optical coherence tomography. J Biomed Opt. 2014; 19:106013. (For interpretation of the references to colour in this figure legend, the reader is referred to the web version of this article.)

With the high-resolution SD-OCT platform described above (Hariri et al., 2014), the CCE hinged flaps at CCE entrances as well as the connections to the TM can be imaged. Resolution is sufficiently high that comparisons can be made between structural features of CCE obtained by SD-OCT and scanning electron microscopy as seen in Fig. 6. CCE typically enter the sclera and then join intrascleral channels in the deep scleral plexus (Fig. 6D and E). These intrascleral channels are typically oriented parallel to SC circumference and have a thin scleral septum separating them from SC (Fig. 6E and F). These parallel intrascleral collector channels also open and close Fig. 6B and C. Systematic steady-state changes in IOP demonstrate that the hinged flaps at CCE and intrascleral collector channels undergo pressure-dependent changes in shape. The shape changes of the hinged flaps are synchronous with changes in the trabecular meshwork configuration (Fig. 6B, C & F). Corresponding changes in lumen dimensions of SC, CCE and parallel intrascleral channels are also apparent.

### 5.2. Ex vivo instantaneous changes of SC and CCE lumen dimensions

**Fig. 7:**
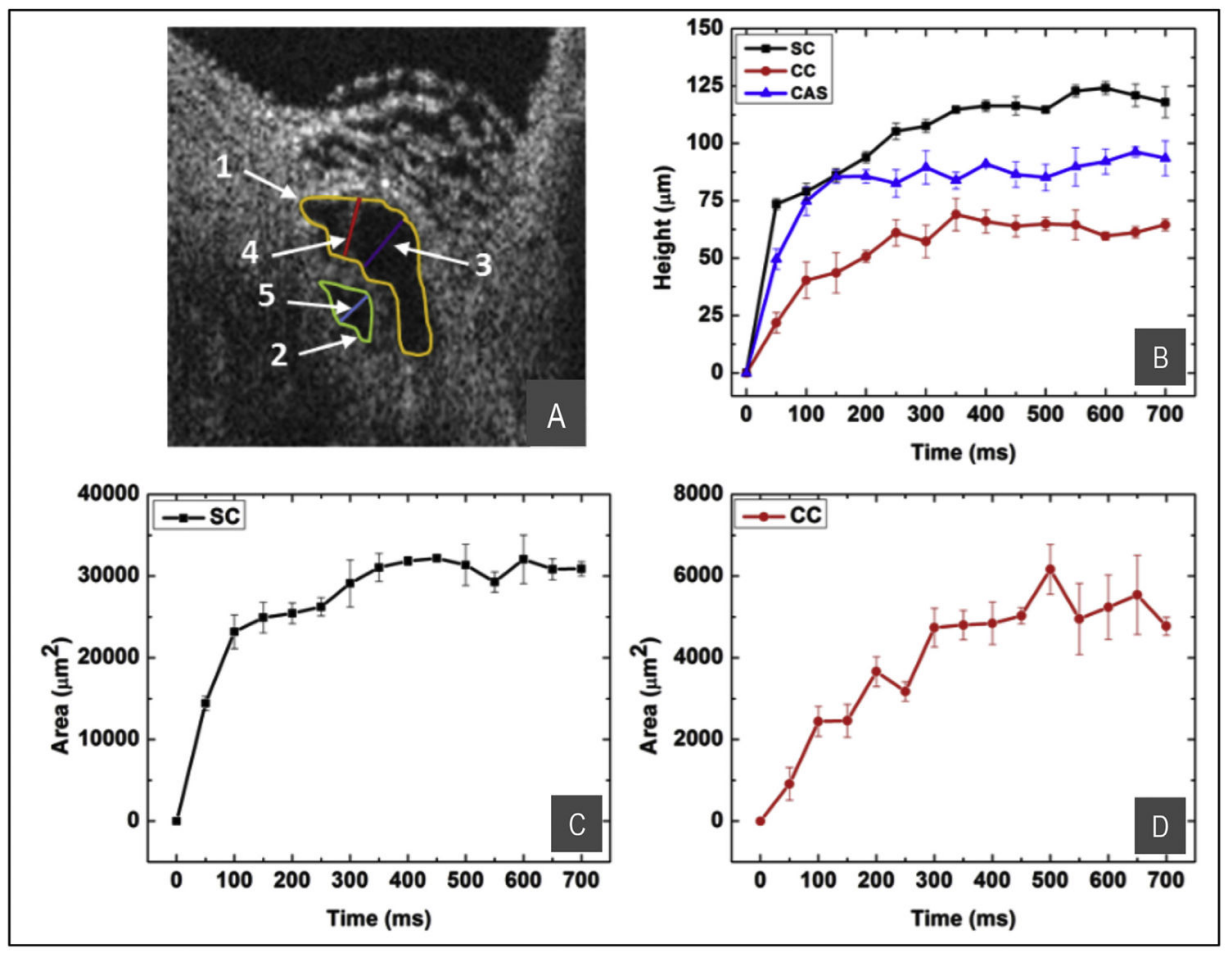
SD-OCT image of SC from Fig. 6F. Parameters for quantification are shown in (A): SC height, purple line; CC height, blue line; CAS height, red line; SC area, yellow line; CC area, green line. (B) Progressive increase in the height of SC (black curve), CC (red curve), and CAS (blue curve). (C) The change in SC lumen area. (D) The change in CC lumen area. Configuration changes rise to reach a plateau within ~100–300 ms. From: Hariri S, Johnstone M, Jiang Y et al. Platform to investigate aqueous outflow system structure and pressure-dependent motion using high-resolution spectral domain optical coherence tomography. J Biomed Opt. 2014; 19:106013.

Pulsed infusion performed while imaging the outflow system permits the measurement of the time course of changes in dimensions of the lumen of SC, of the CCE and of cylindrical attachment structures connecting the TM and CCE (Fig. 7) (Hariri et al., 2014). Changes in SC lumen dimensions are reflective of changes in the TM configuration as it moves away from SC external wall. CCE lumen dimension changes reflect changes in the orientation of hinged flaps at CCE entrances. The height and area of both the SC and CCE lumen increase from a closed configuration at 0 mm Hg IOP to plateau at a wide-open configuration within less than 300 ms. The time course of the motion of the TM and CCE is synchronous and the cylindrical attachment structures connecting the TM and flaps at CCE move with the same time course.

## 6. *Ex vivo* PhS-OCT platform images pressure-dependent TM motion

### 6.1. The technology

PhS-OCT, first developed in 2007, uses the phase changes between adjacent B-scans to measure tissue movement (Wang et al., 2007a, 2007b). PhS-OCT has extremely high sensitivity to motion being able to measure motion of as little as 0.26 nm. PhS-OCT permits transcleral detection of TM tissue movement because of the dynamic range conferred by its nanoscale sensitivity to motion. In contrast, SD-OCT systems, as a result of limitations discussed in Section 4.1, are unable to measure real-time pulse induced motion of the trabecular tissues through the sclera.

Although PhS-OCT cannot directly image structure, two strategies permit correlating the functional data related to motion with structural information. 1) PhS-OCT and SD-OCT information is obtained from the same dataset permitting registration and overlay of phase data onto structural images. 2) Regions with phase motion can be segmented out and the background subtracted leaving an outline that uses functional properties to define anatomic structures as is done in OCT-based label-free angiography.

### 6.2. Ex vivo PhS-OCT imaging of pressure-dependent TM motion

**Fig. 8:**
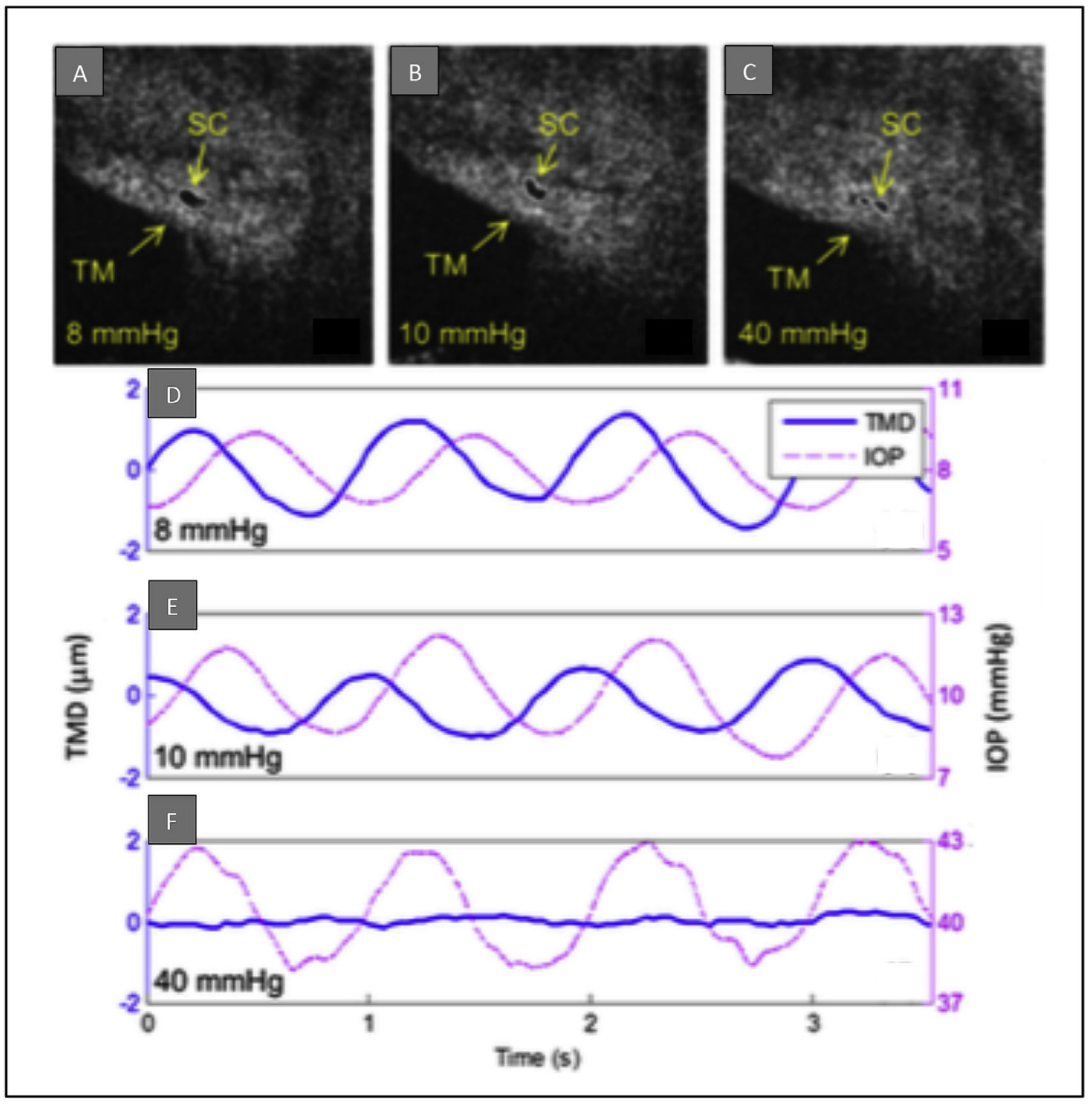
IOP-dependence of SC deformation (SD-OCT images) and TM movement (PhS-OCT measurements) derived from the same data set in *ex vivo* whole eye primate experiments. Images of the same SC cross-sections at an intraocular pressure (IOP) of 8 (A),10 (B), and 40 (C) mm Hg, respectively. Fig. D to F images are corresponding plots of IOP (dashed red lines) and TM displacement (blue lines) versus time at each IOP. The TM mean displacement (TMD) is markedly reduced with the increase of the mean IOP. From: Li P, Reif R, Zhi Z et al. Phase-sensitive optical coherence tomography characterization of pulse-induced trabecular meshwork displacement in *ex vivo* nonhuman primate eyes. J Biomed Opt. 2012; 17:076026.

**Fig. 9:**
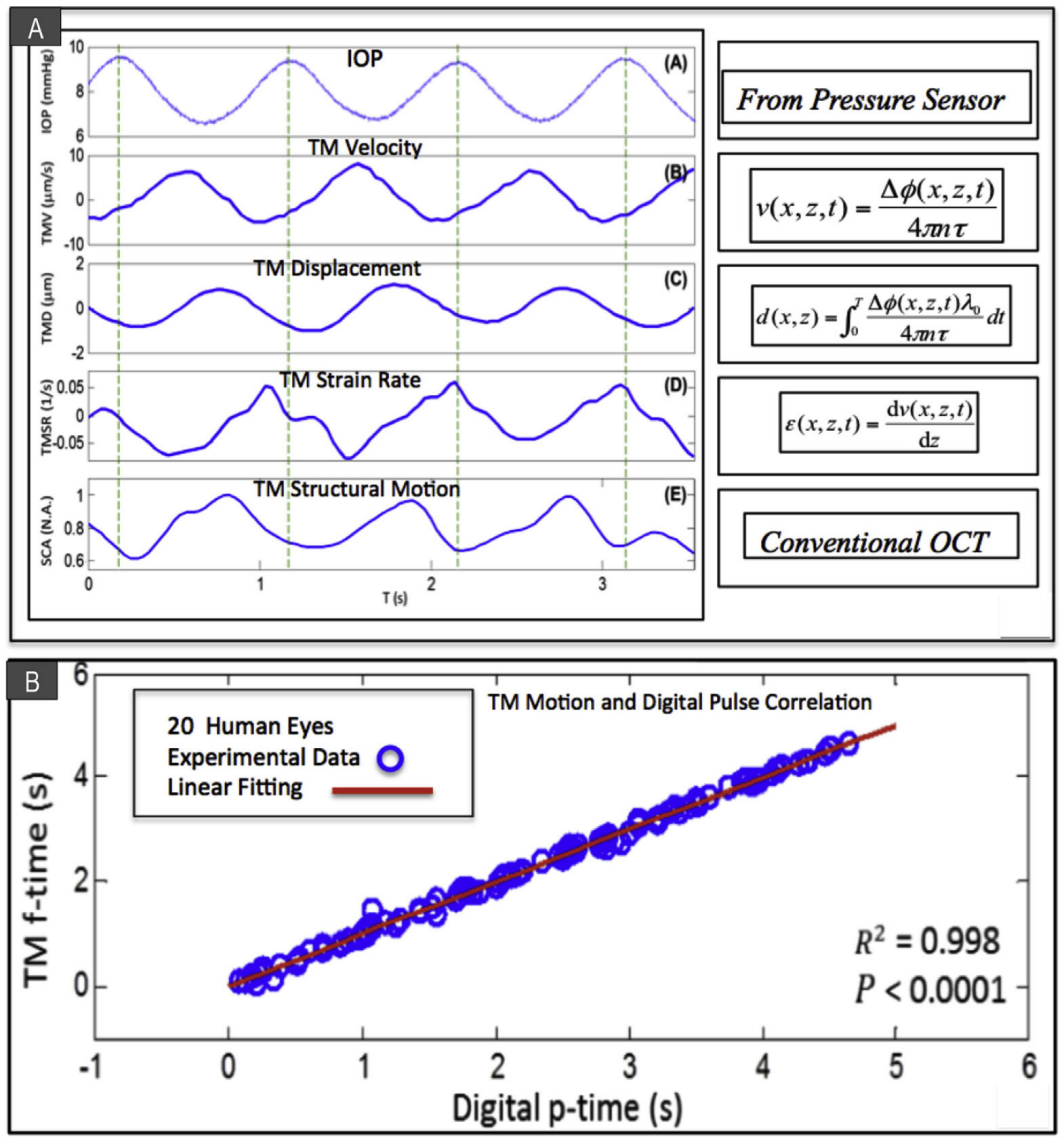
(A) Ex vivo PhS-OCT whole eye experiments. Temporal plots over 3.5 s of IOP measurements provided by an in-line pressure transducer synchronized with PhS-OCT system. (inset (A)) IOP; (inset (B)) trabecular meshwork velocity (TMV); (inset (C)) trabecular meshwork displacement (TMD); (Inset(D)) trabecular meshwork strain rate (TMSR) and (inset (E)) normalized SC size (SCA). The experimental conditions were mean IOP 8 mmHg, pulse amplitude 3 mmHg and 1 pulse/second. The dashed vertical lines indicate the time of the IOP pulse peaks. (B) In vivo PhS-OCT in human subjects. Arrival times of human digital pulse vs. TM motion peak. A significant correlation (R^2^ = 0.998, P < 0.0001) of the instantaneous time between the digital pulse peaks [digital p-time, marked by black thin arrows in Fig. 3(D)] and the TM pulse minima [TM f-time, marked by blue bold arrows in Fig. 3(D)] is found. The results demonstrate the temporal synchronization between the cardiac pulse and the TM motion. (A) from: Li P, Reif R, Zhi Z et al. Phase-sensitive optical coherence tomography characterization of pulse-induced trabecular meshwork displacement in *ex vivo* nonhuman primate eyes. J Biomed Opt. 2012; 17:076026. (B) from: Li P, Shen TT, Johnstone M, Wang RK. Pulsatile motion of the trabecular meshwork in healthy human subjects quantified by phase-sensitive optical coherence tomography. Biomed Opt Express. 2013; 4:2051–2065. (For interpretation of the references to colour in this figure legend, the reader is referred to the web version of this article.)

Perfusion of whole enucleated NH primate eyes from a reservoir system controlled mean IOP while inducing sinusoidal pulse transients (Li et al., 2012) (Fig. 8). Measurements were carried out at a series of seven different mean pressures while maintaining pulse amplitude of 3 mm Hg and frequency of 1 pulse/second (1 Hz). The study demonstrated TM motion synchronous with pulse-induced IOP transients. SC lumen size decreased as IOP increased (Fig. 8A, B & C). Pulsatile TM motion also decreased as IOP increased and was absent at 40 mm Hg (Fig. 8D, E & F). Quantitative measurements in this *ex vivo* model included velocity, displacement, strain rate, and TM structural motion (Fig. 9A) parameters important for assessing the biomechanical properties of tissues.

## 7. *In vivo* PhS-OCT platform images pulse-dependent TM motion in humans

PhS-OCT has recently succeeded in detecting pulse induced TM motion *in vivo* in twenty eyes of 10 human subjects (Li et al., 2013) (Fig. 9B). The key to this success is the development of a phase compensation algorithm that permits removal of the confounding effects of bulk motion. A correlation between the TM motion and the digital pulse was found to be highly significant (P < 0.0001). The digital pulse led the TM motion by a mean time of 0.5 ± 0.48 s. A significant linear relationship was present between the phase lag and the pulse rate (P<. 05). Phase lag was affected by age but did not quite reach significance (P = 0.074).

**Fig. 10:**
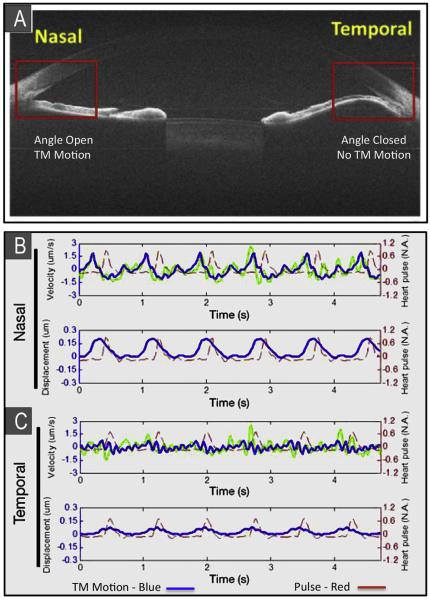
(**A**) Phase-sensitive optical coherence tomography (PhS-OCT) assessment of dynamic motion of TM of a human subject *in vivo*. The nasal anterior chamber (AC) angle is normal. An iris cyst closes the temporal angle preventing access of aqueous to the temporal outflow system. OCT cross-sectional image size = 12 mm × 10 mm. (**B**) The pulse tracing is in red. Velocity and displacement tracings (in blue) of the trabecular meshwork (TM) motion in the normal nasal angle are easily identified and are synchronous with the peripheral pulse. (C) TM velocity and amplitude tracings in the temporal closed angle are barely discernable. From: Y S, P L, Johnstone M, K W, T S. Pulsatile motion of trabecular meshwork in a patient with iris cyst by phase-sensitive optical coherence tomography. Quantitative Imaging in Medicine and Surgery. 2015; 5:171–173.

Comparison of normal and abnormal outflow system regions in the same eye of a patient (Fig. 10) was made possible because a unilateral primary iris cyst closed the angle on the temporal side of the eye while the nasal angle remained wide open (Sun et al., 2015). Because the iris cyst completely occluded the temporal angle, that region of the outflow system did not communicate with the anterior chamber. In the normal nasal angle TM tracings of velocity and displacement amplitude were relatively large and synchronous with the digital pulse. In the occluded temporal angle, TM velocity and amplitudes were barely discernable.

## 8. Aqueous outflow system OCT imaging in the management of glaucoma

### 8.1. Current status

Kagemann et al. (2014) achieved an important milestone in outflow imaging in humans by demonstrating a decrease in SC area in response to an increase in IOP using a commercially available SD-OCT system. However, the resolution of current commercial systems is insufficient to make them an easily used clinical tool for monitoring outflow system motion.

Laboratory-based PhS-OCT is capable of imaging pulse-dependent TM motion from the scleral surface but would require the involvement of industry to develop a commercial system for external use or a probe for direct observation through the TM. The laboratory-based PhS-OCT system described in this review demonstrates that such a system with its high degree of sensitivity to motion can image TM pulse-dependent behavior from the scleral surface in human subjects.

Laboratory-based OCT studies also show that the TM and CCE can be imaged from the TM surface and their motion quantified with an optimized system. The laboratory technique of pressure reversal that allows detailed study of TM and CCE motion and relationships is already used in the operating room to predict the probability of surgical success (see Section 4.0). The imaging capabilities of a trans-trabecular OCT system as described above suggest that development of an OCT probe would be very useful in a surgical environment to assess and target areas with CCE to determine the functional status of the distal outflow system.

Circumferential flow around SC has been demonstrated to be quite limited (Grant, 1958). For that reason placement of a MIGS device at a single location can be expected to provide access to only a small area of SC circumference and few CCE. Studies also show that aqueous outflow is not distributed equally around the 360° circumference of SC (Johnstone, 2006). If CCE and distal outflow pathways are nonfunctional at the chosen location for device insertion the procedure can be expected to fail. Use of OCT technology may permit identifying regions of the trabecular meshwork and CCE that experience pulse-induced motion. Pulse-induced motion is indicative of a region with mobile tissues capable of changing shape to accommodate aqueous flow. Locations with active motion and aqueous flow also suggest the presence of a functional distal outflow system that may be an optimal target for MIGS placement.

### 8.2. Future imaging possibilities

PhS-OCT is non-contact, non-invasive and measures tissue properties associated with maintaining IOP homeostasis. Research studies using *in vivo* slitlamp and gonioscopic imaging identify defective outflow system motion in humans with glaucoma. Defects include both reduced/absent aqueous vein pulsation (Section 2.2) and lack of TM motion (Section 2.3). The clinical techniques currently used to identify defective TM motion are sufficiently laborious and time consuming that they have not been widely used for detection of outflow system abnormalities in a clinical environment. However non-invasive techniques that can image outflow system function in the office should prove to be highly useful for both detecting and monitoring abnormalities that result in pressure elevation.

The current use of random IOP measurements to detect and monitor glaucoma is problematic because the approach captures only a small part of the IOP profile. Pressures undergo diurnal changes and also numerous transient IOP elevations from baseline in the order of 10 mm Hg associated with blinking and eye movement. IOP measurements are typically made randomly 3–4 times per year representing sampling of ~12 s of the 31 million seconds in the year. Diurnal pressures vary considerably and often rise markedly at night when no measurements are made. Measurements are also made while specifically avoiding blinking or eye movement thus preventing capture of any of the constantly recurring transient IOP elevations. Furthermore, medication compliance unknowns are a confounding factor further clouding the assessment of the true pressure profile.

Motion of the TM in response to the ocular pulse is indicative of elasticity and compliance of the trabecular tissues, properties that determine IOP homeostasis. Knowledge of TM motion revealed by OCT imaging may permit identifying individual patients who are experiencing progressive difficulty in maintaining homeostasis not easily revealed by random IOP measurements. Such imaging may provide a sensitive predictive tool to help decide if preemptive escalation of medical, laser or surgical therapy is appropriate before the patient develops the late warning of progressive structural and functional damage to the visual system.

The ability to assess motion with PhS-OCT may improve selection of candidates for SC surgery. By eliminating inappropriate candidates, patients would be spared unnecessary surgery. At the same time procedure success rates as a result of appropriate selection might be considerably higher. Blood reflux into SC while in the operating room has been shown to be a predictor of the likelihood of SC surgical success. Blood reflux into SC depends on the ability of the TM to move. Use of a probe inserted inside the eye to image through the TM may provide high-resolution images to identify CCE motion indicative of a functional region of the distal outflow system.

The optimal placement of MIGS devices might be substantially improved if functioning CCE could be clearly identified. Regions where CCE are permanently closed or that have inadequate motion could be avoided. Patients would be spared from MIGS surgery when CCE dysfunction indicates a limited possibility for success and could instead move directly to more appropriate alternatives.

## 9. Summary

*In vivo* clinical studies are capable of using imaging to define aqueous outflow system abnormalities in glaucoma. These studies point to an abnormality of trabecular tissue motion that prevents normal pulsatile aqueous outflow and also prevents blood reflux into SC. Such motion abnormalities have been thought to result from a stiffening of trabecular tissues. The available clinical techniques are difficult and time consuming.

A recently developed high resolution SD-OCT platform that images the outflow system from the TM surface identifies hinged flaps at CCE. The hinged flaps open and close in synchrony with pulse-dependent TM motion. Another recently developed PhS-OCT technology images pulse-dependent TM motion in human subjects. Rapidly evolving OCT imaging techniques may develop into predictive clinical tools that will assist in the medical and surgical management of glaucoma.
